# Cdc20 Is Critical for Meiosis I and Fertility of Female Mice

**DOI:** 10.1371/journal.pgen.1001147

**Published:** 2010-09-30

**Authors:** Fang Jin, Masakazu Hamada, Liviu Malureanu, Karthik B. Jeganathan, Wei Zhou, Dean E. Morbeck, Jan M. van Deursen

**Affiliations:** 1 Department of Pediatric and Adolescent Medicine, Mayo Clinic College of Medicine, Rochester, Minnesota, United States of America; 2 Department of Biochemistry and Molecular Biology, Mayo Clinic College of Medicine, Rochester, Minnesota, United States of America; 3 Department of Obstetrics and Gynecology, Mayo Clinic College of Medicine, Rochester, Minnesota, United States of America; National Institute of Diabetes and Digestive and Kidney Diseases, United States of America

## Abstract

Chromosome missegregation in germ cells is an important cause of unexplained infertility, miscarriages, and congenital birth defects in humans. However, the molecular defects that lead to production of aneuploid gametes are largely unknown. Cdc20, the activating subunit of the anaphase-promoting complex/cyclosome (APC/C), initiates sister-chromatid separation by ordering the destruction of two key anaphase inhibitors, cyclin B1 and securin, at the transition from metaphase to anaphase. The physiological significance and full repertoire of functions of mammalian Cdc20 are unclear at present, mainly because of the essential nature of this protein in cell cycle progression. To bypass this problem we generated hypomorphic mice that express low amounts of Cdc20. These mice are healthy and have a normal lifespan, but females produce either no or very few offspring, despite normal folliculogenesis and fertilization rates. When mated with wild-type males, hypomorphic females yield nearly normal numbers of fertilized eggs, but as these embryos develop, they become malformed and rarely reach the blastocyst stage. In exploring the underlying mechanism, we uncover that the vast majority of these embryos have abnormal chromosome numbers, primarily due to chromosome lagging and chromosome misalignment during meiosis I in the oocyte. Furthermore, cyclin B1, cyclin A2, and securin are inefficiently degraded in metaphase I; and anaphase I onset is markedly delayed. These results demonstrate that the physiologically effective threshold level of Cdc20 is high for female meiosis I and identify Cdc20 hypomorphism as a mechanism for chromosome missegregation and formation of aneuploid gametes.

## Author Summary

Aneuploidy, an abnormal number of chromosomes, is a common defect in sperm and egg cells that is responsible for human infertility, miscarriage, and congenital birth defects. Although these developmental outcomes are prevalent in human reproduction, little is known about the molecular defects that may cause aneuploidy in germ cells. In this study, we identify Cdc20, a critical activator of the APC/C E3 ubiquitin ligase that initiates sister chromosome separation by ordering the destruction of cyclin B1 and securin, as a female infertility gene. We show that female mice with low amounts of Cdc20 have normal fitness but almost exclusively produce aneuploid embryos that fail to thrive and die early in development. The aneuploidy primarily results from chromosome segregation errors in primary oocytes that may be caused by inefficient APC/C-mediated destruction of mitotic cyclins and securin during metaphase I. Thus, our studies reveal that primary oocytes are highly dependent on Cdc20 for accurate chromosome segregation and raise the possibility that Cdc20 insufficiency may be a cause of infertility in otherwise healthy women.

## Introduction

Mitotic checkpoint genes are believed to be prime targets for deregulation in human infertility [1]. The mitotic checkpoint constitutes an intricate molecular network that ensures accurate chromosome segregation by coordinating metaphase-to-anaphase progression with the establishment of bipolar spindle attachment and metaphase plate alignment of all mitotic chromosome pairs [2]. At early stages of mitosis, various mitotic checkpoint proteins, including members of the Bub and Mad protein families, concentrate at unattached kinetochores to generate a diffusible signal that inhibits the anaphase-promoting complex or cyclosome (APC/C), a large E3 ubiquitin ligase that drives metaphase-to-anaphase transition by catalyzing the ubiquitination and degradation of cyclin B1 and securin [3]. Although the exact composition of the inhibitory signal remains a major subject of investigation, it is believed to contain Bub3-bound BubR1 and Mad2 that has been primed by kinetochore-associated Mad1-Mad2 to stably interact with the APC/C activating subunit Cdc20 [4], [5], [6]. Upon attachment and alignment of the last chromosome pair, the inhibitory signal is quenched and APC/C activated through release of Cdc20 inhibition, triggering the ubiquitination and destruction of cyclin B1 and securin. Separase, a protease that is held in an inactive state by securin and cyclin B1/Cdk1, is then allowed to cleave the Scc1 subunit of the cohesin complex that holds sister chomatids together, inducing the physical separation of sister chromatids by spindle forces [7], [8].

A thorough assessment of the role of mitotic checkpoint genes in gametogenesis and infertility has not been possible because complete inactivation of mammalian mitotic checkpoint genes invariably disrupts the chromosome segregation process so severely that cells cannot survive [2], [9]. In vitro studies of primary mouse oocytes in which key mitotic checkpoint proteins were depleted by morpholinos or RNA interference have pointed to an importance of several mitotic checkpoint proteins during the first meiotic division. For instance, sustained prophase I arrest of primary oocytes depends on stabilization of the Cdc20-related APC/C coactivator Cdh1 by BubR1 [10]. BubR1 retains control of Cdh1 stability after hormone-induced resumption of meiosis, thereby allowing APC/C^Cdh1^-mediated securin degradation and progression through prometaphase I. Interestingly, BubR1 protein levels have been shown to decline in ovary and testis as normal mice age, which combined with the observation that mutant mice with low amounts of BubR1 are infertile, has led to speculation that BubR1 might be a key determinant of age-related meiotic errors in germ cells [11]. While APC/C^Cdh1^ regulates early meiotic events in mice [10], [12], Cdc20 knockdown experiments in primary oocytes indicate that APC/C^Cdc20^ is active in late meiosis I [10], where it is responsible for driving oocytes into anaphase via the destruction of cyclin B1and securin, much like mitosis in somatic cells [13]. Coordination of APC/C^Cdc20^ activation with proper kinetochore-microtubule attachment in meiosis I is dependent on the mitotic checkpoint proteins Mad2 and Bub1, as depletion or expression of dominant-negative mutants of these proteins in primary mouse oocytes causes chromosome missegregation [14], [15], [16], [17].

Whereas the depletion studies in primary mouse oocytes identify Cdc20 and Cdh1 as critical regulators of the first meiotic division, testing whether the functions unveiled in vitro operate in vivo remains an important challenge. Furthermore, it remains unknown whether Cdc20 and Cdh1 are also important for male meiosis I or stages of male and female gametogenesis other than meiosis I. Importantly, for Cdc20 and Cdh1 to be candidate infertility genes, one would expect their dysfunction to reduce fertility without compromising overall health and viability. Addressing these issues has been hampered by the embryonic lethality caused by inactivation of *Cdh1* and *Cdc20* in mice, with *Cdh1*-null embryos dying at mid-gestation due to placental defects [18], [19] and *Cdc20*-null embryos at the two-cell stage due to permanent metaphase arrest [18].

In the present study, we bypassed the problem of early embryonic lethality of *Cdc20* knockout mice by generating mutant mouse strains in which the dose of Cdc20 is reduced in graded fashion, enabling us to examine the physiological relevance of this APC/C cofactor. Our findings reveal that the threshold for pathophysiology is lowest in the female germline. We demonstrate that while both mitotic and meiotic divisions of male and female germ cells are characterized by inaccurate chromosome segregation and aneuploidization, only female meiosis I is so severely affected that almost exclusively aneuploid mature eggs are generated. We show that these eggs fertilize normally, but that the resulting zygotes die after the first few embryogenic divisions.

## Results

### Generation of Mutant Mice with Graded Reduction of Cdc20

**Figure 1:**
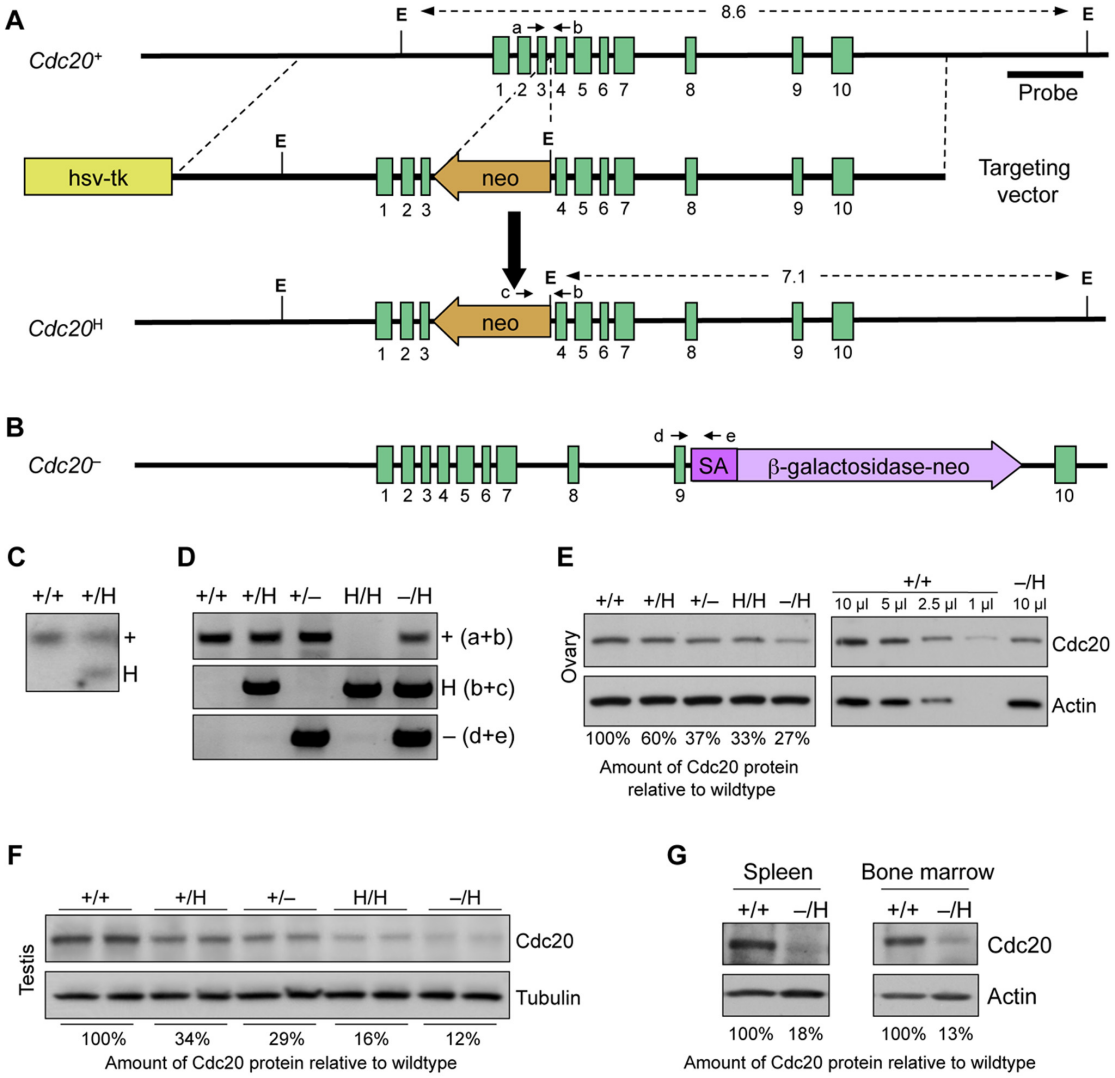
Generation of mice with graded reduction in Cdc20 dosage. (A) Schematic representation of the primary Cdc20 gene targeting strategy. Part of the Cdc20 locus (+), the targeting vector, the hypomorphic allele (*Cdc20* ^H^), *EcoR1* restriction sites and the Southern probe are indicated. (B) Schematic representation of the *Cdc20* ^−^allele was from gene trap mouse embryonic stem (ES) cell clone XE368. (C) Southern-blot analysis of mice with indicated *Cdc20* genotypes. (D) PCR-based genotype analysis of *Cdc20* mutant mice. Positions of PCR primers (a–e) are indicated in (A,B). (E–G) Western blot analysis of whole ovary (E), testis (F), spleen and bone marrow (G) extracts of the indicated genotypes for Cdc20. Actin and tubulin served as loading controls. Cdc20 protein signals were quantified using ImageJ software and normalized to background and either actin or tubulin. For details see materials and methods.

A series of mutant mouse strains in which expression of Cdc20 is gradually reduced was generated by using various combinations of wild-type (*Cdc20* ^+^), hypomorphic (*Cdc20* ^H^) and knockout (*Cdc20* ^−^) alleles (Figure 1A–1D). The *Cdc20* ^H^ allele was produced by targeted insertion of a neomycin phosphotransferase II (neo) gene cassette into the third intron of the *Cdc20* gene (Figure 1A). The neo gene contains a cryptic exon with stop codons in all three reading frames, thereby considerably reducing the amount of wild-type protein produced by targeted allele [11], [20], [21], [22]. The *Cdc20* ^−^ allele was from gene trap mouse embryonic stem (ES) cell clone XE368 (Figure 1B). Previously, it has been shown that this gene trap allele is the equivalent of a null allele and that embryos that are homozygous for this allele arrest and die at the two-cell stage of development [23]. In contrast, *Cdc20* ^+/H^, *Cdc20* ^+/−^, *Cdc20* ^H/H^ and *Cdc20* ^−/H^ mice were viable and had no overt phenotypes. Western blot analysis demonstrated that *Cdc20* ^+/H^, *Cdc20* ^+/−^, *Cdc20* ^H/H^ and *Cdc20* ^−/H^ ovary and testes had a graded reduction of Cdc20 protein (Figure 1E and 1F). Western blot analysis of spleen, bone marrow, and mouse embryonic fibroblast extracts of *Cdc20* ^+/+^ and *Cdc20* ^−/H^ mice suggested that the observed Cdc20 protein reductions are universal, irrespective of tissue or cell type (Figure 1G, and data not shown).

### Cdc20 Hypomorphic Females Are Infertile or Subfertile Despite Normal Oogenesis

**Figure 2:**
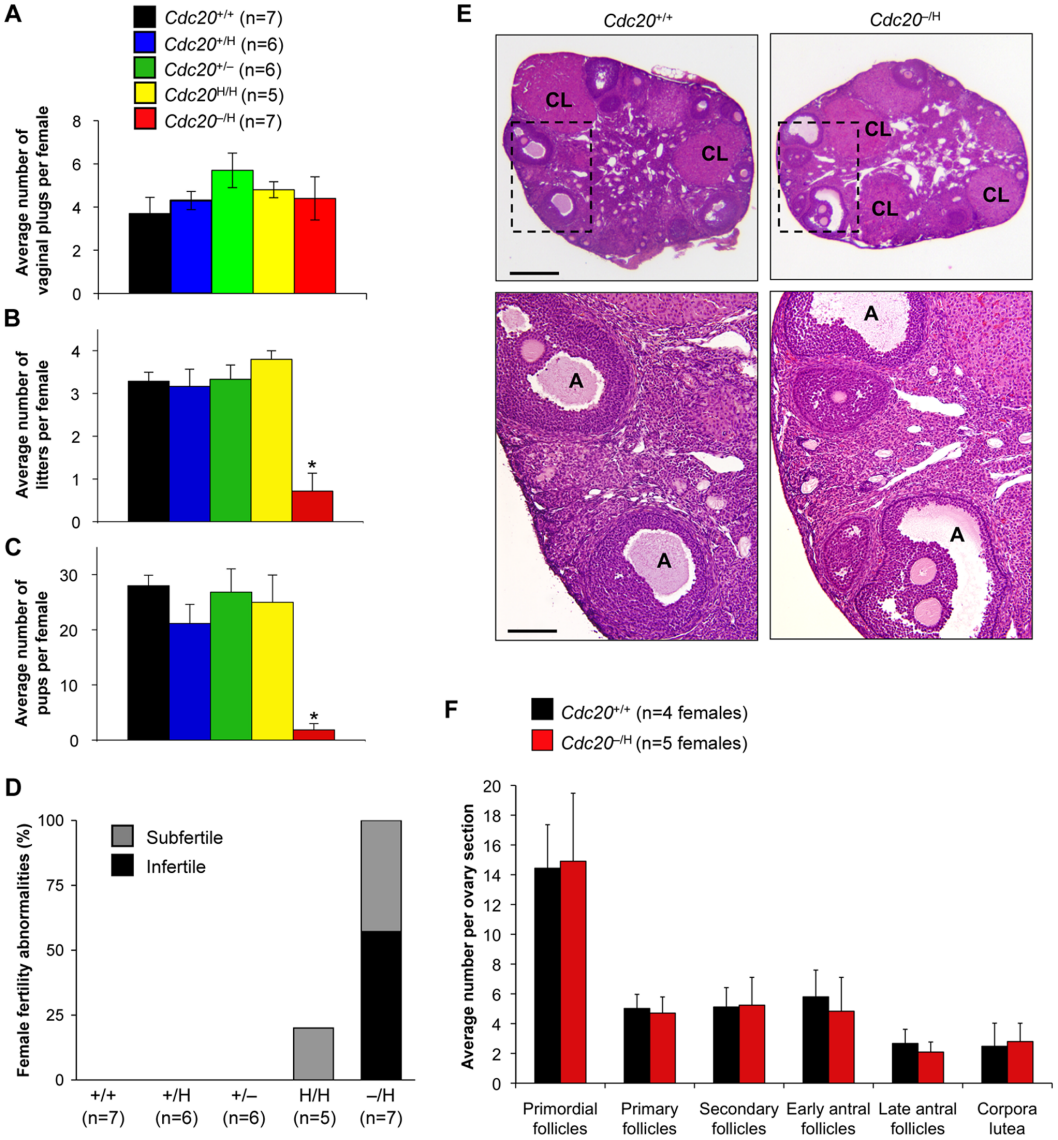
Female mice with low amounts of Cdc20 have poor fertility. (A) Average number of vaginal plugs per female for the indicated genotypes (during 3 months of breeding). (B) Average number of litters per female for the indicated genotypes (during 3 months of breeding). Data presented in (A,B) are mean ± SEM. (C) Average number of pups per female (during 3 months of breeding). Chart legend is as in (A). Asterisks indicate statistical significance (one way ANOVA p<0.0001) between *Cdc20* ^−/H^ and the other genotypes. (D) Percentages of subfertile and infertile females per genotype. (E) H/E-stained ovary sections from *Cdc20* ^+/+^ and *Cdc20* ^−/H^ females (5 µm paraffin sections). A =  antral follicles; CL =  corpus luteum. Bars in top and bottom panels are 400 µm and 100 µm, respectively. (F) Quantification of various follicles and corpora lutea in H/E sections of *Cdc20* ^+/+^ and *Cdc20* ^−/H^ ovaries. Error bars represent mean ± SEM.

While establishing cohorts of Cdc20 mutant mice for long-term observation, we noticed that *Cdc20* ^−/H^ females yielded little or no offspring, which prompted us to measure the impact of graded reduction in Cdc20 expression on female fertility. Two-month-old *Cdc20* ^+/+^, *Cdc20* ^+/H^, *Cdc20* ^+/−^, *Cdc20* ^H/H^ and *Cdc20* ^−/H^ mice were bred to *Cdc20* ^+/+^ males of the same age and the number of litters and pups produced per female was recorded for three months. Despite normal copulation rates (Figure 2A), *Cdc20* ^−/H^ females produced on average about 4-fold fewer litters than females of the other genotypes (Figure 2B), while the average number of pups was about 15-fold lower (Figure 2C). Notably, of the seven *Cdc20* ^−/H^ females in the study, four failed to produce any offspring (Figure 2D). Only *Cdc20* ^+/−^ and *Cdc20* ^+/H^ embryos can be produced by *Cdc20* ^−/H^ females bred to *Cdc20* ^+/+^ males. Importantly, pups of these genotypes were produced at normal rates when *Cdc20* ^+/−^, *Cdc20* ^+/H^ and *Cdc20* ^H/H^ females were bred to *Cdc20* ^+/+^ males (Figure 2C and 2D), indicating that the failure of *Cdc20* ^−/H^ females to produce offspring with *Cdc20* ^+/+^ males was not due to the genotype of the embryos produced. Together, the above data demonstrate that *Cdc20* ^−/H^ females are either infertile or severely subfertile. The Cdc20 threshold level for fertility problems is remarkably sharp because *Cdc20* ^H/H^ females, which produce slightly more Cdc20 than *Cdc20* ^−/H^ females, have normal fertility (Figure 2A–2D). Ten of 10 *Cdc20* ^−/H^ males were fertile and produced on average 7 pups per litter (data not shown), indicating that gametogenesis in male mice has a lower dependence on Cdc20 than the female reproductive system.

To study how Cdc20 deficiency impedes female fertility, we screened hematoxylin-eosin ovary sections of sexually mature *Cdc20* ^−/H^ females for overt defects in oogenesis. However, no apparent morphological differences were found (Figure 2E). *Cdc20* ^−/H^ and *Cdc20* ^+/+^ ovary sections contained similar amounts of primordial, primary, secondary and antral follicles, as well as similar numbers of mature oocytes and corpora lutea (Figure 2E and 2F). These data indicated that the fertility problem of *Cdc20* ^−/H^ females is not due to a failure to produce, mature or ovulate oocytes.

### Fertilized Eggs from Cdc20 Hypomorphic Females Fail to Develop into Blastocysts

**Figure 3:**
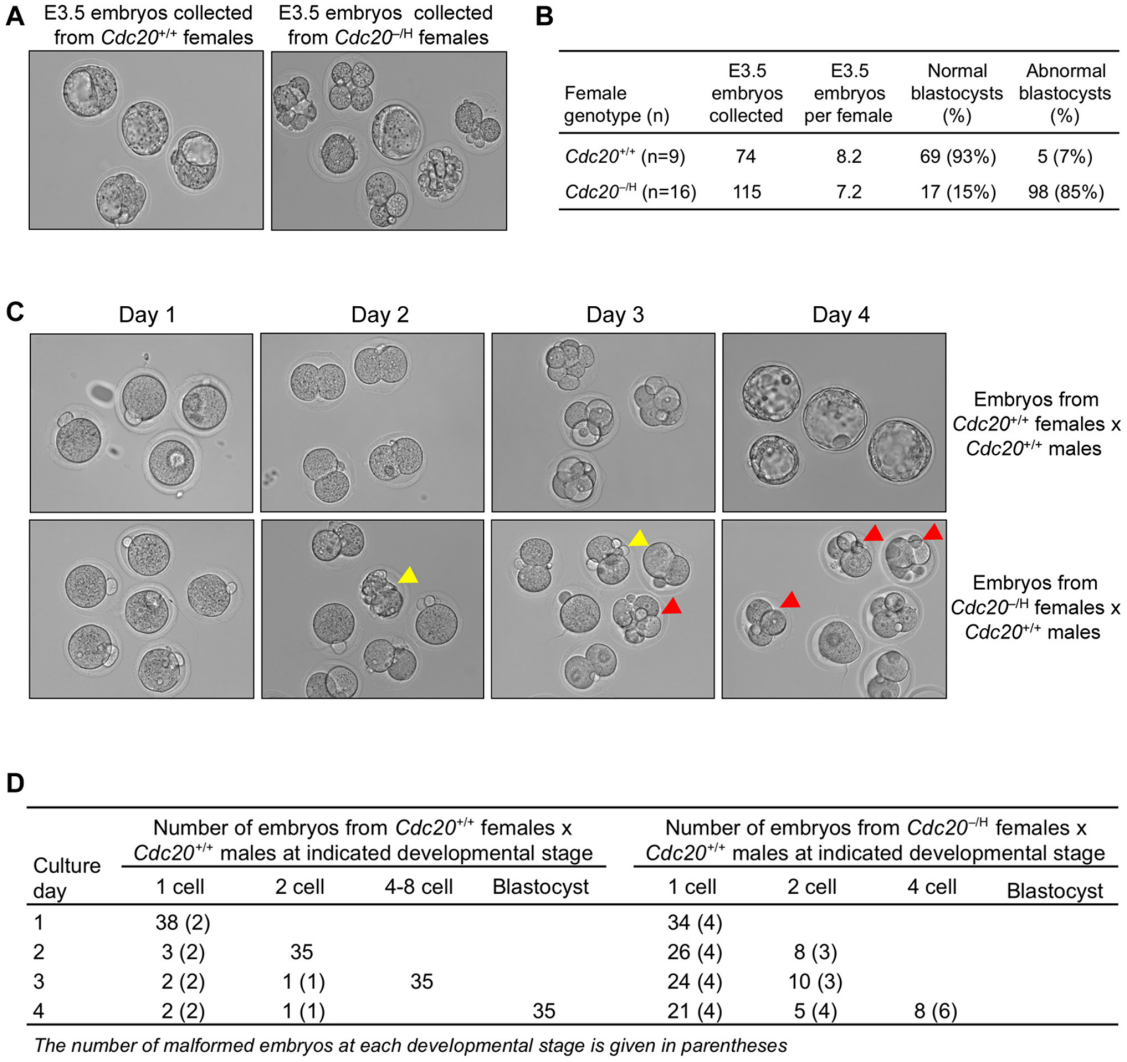
*Cdc20* ^−/H^ eggs fertilized by *Cdc20* ^+/+^ males rarely develop into blastocysts. (A) In vivo development of E3.5 embryos from *Cdc20* ^+/+^ and *Cdc20* ^−/H^ females crossed with *Cdc20* ^+/+^ males. (B) Quantitation of the in vivo developmental defects. (C) In vitro development of E0.5 embryos from *Cdc20* ^+/+^ and *Cdc20* ^−/H^ females fertilized by *Cdc20* ^+/+^ males. Images were collected at 24 h intervals (day 1 is the day of embryo collection, which corresponds to E0.5). Note that none of the embryos from *Cdc20* ^−/H^ females developed beyond the 4-cell stage. Yellow and red arrowheads highlight malformed two- and four-cell stage embryos, respectively. (D) Quantitation of the in vitro developmental defects. Note that 62% of the embryos from *Cdc20* ^−/H^ females crossed with *Cdc20* ^+/+^ males failed to develop beyond the one-cell stage.

To explore preimplantation embryonic development, *Cdc20* ^−/H^ and *Cdc20* ^+/+^ females were naturally mated with *Cdc20* ^+/+^ males and embryos were collected at day 3.5 of development (E3.5). While 93% of embryos collected from *Cdc20* ^+/+^ females were at the expected blastocyst stage, only 15% of *Cdc20* ^−/H^ females had reached this stage (Figure 3A and 3B). The remaining embryos were either in the one- to four-cell stage or completely degenerated. Notably, the total number of embryos produced by *Cdc20* ^+/+^ and *Cdc20* ^−/H^ females was the same (Figure 3B), indicating *Cdc20* ^−/H^ females had normal fertilization rates and were capable of ovulating normal numbers of mature oocytes. Furthermore, the number of normal blastocysts produced by *Cdc20* ^−/H^ females is similar to the number of live born pups these females produce, indicating that embryos that attain the blastocyst stage were capable of developing into healthy animals.

The above data indicated that the majority of eggs produced by *Cdc20* ^−/H^ females stop proliferating after the first cell divisions of the preimplantation period. To confirm this and to characterize preimplantation embryo development, we collected one-cell stage embryos from *Cdc20* ^+/+^ and *Cdc20* ^−/H^ females crossed with *Cdc20* ^+/+^ males and monitored their development in vitro. As expected, most embryos from *Cdc20* ^+/+^ females developed to the blastocyst stage within four days (Figure 3C and 3D). In contrast, none of the embryos from *Cdc20* ^−/H^ females developed beyond the 4-cell stage, with the majority of embryos remaining at the one cell stage. This growth phenotype is remarkably different from that of *Cdc20* ^−/−^ embryos, which typically arrest in metaphase at the two-cell stage due to inability to degrade cyclin B1 and securin in the absence of Cdc20 [23]. Importantly, one cell stage embryos from *Cdc20* ^−/H^ females are either *Cdc20* ^+/−^ or *Cdc20* ^+/H^. Embryos of these genotypes show normal survival rates when derived from *Cdc20* ^+/−^ and *Cdc20* ^+/H^ females and *Cdc20* ^+/+^ males (see Figure 2B and 2C). Together, these data suggested that the early death of the embryos produced by *Cdc20* ^−/H^ females is due to defects introduced during oogenesis.

### Cdc20 Hypomorphic Females Produce Aneuploid Oocytes and Embryos

**Figure 4:**
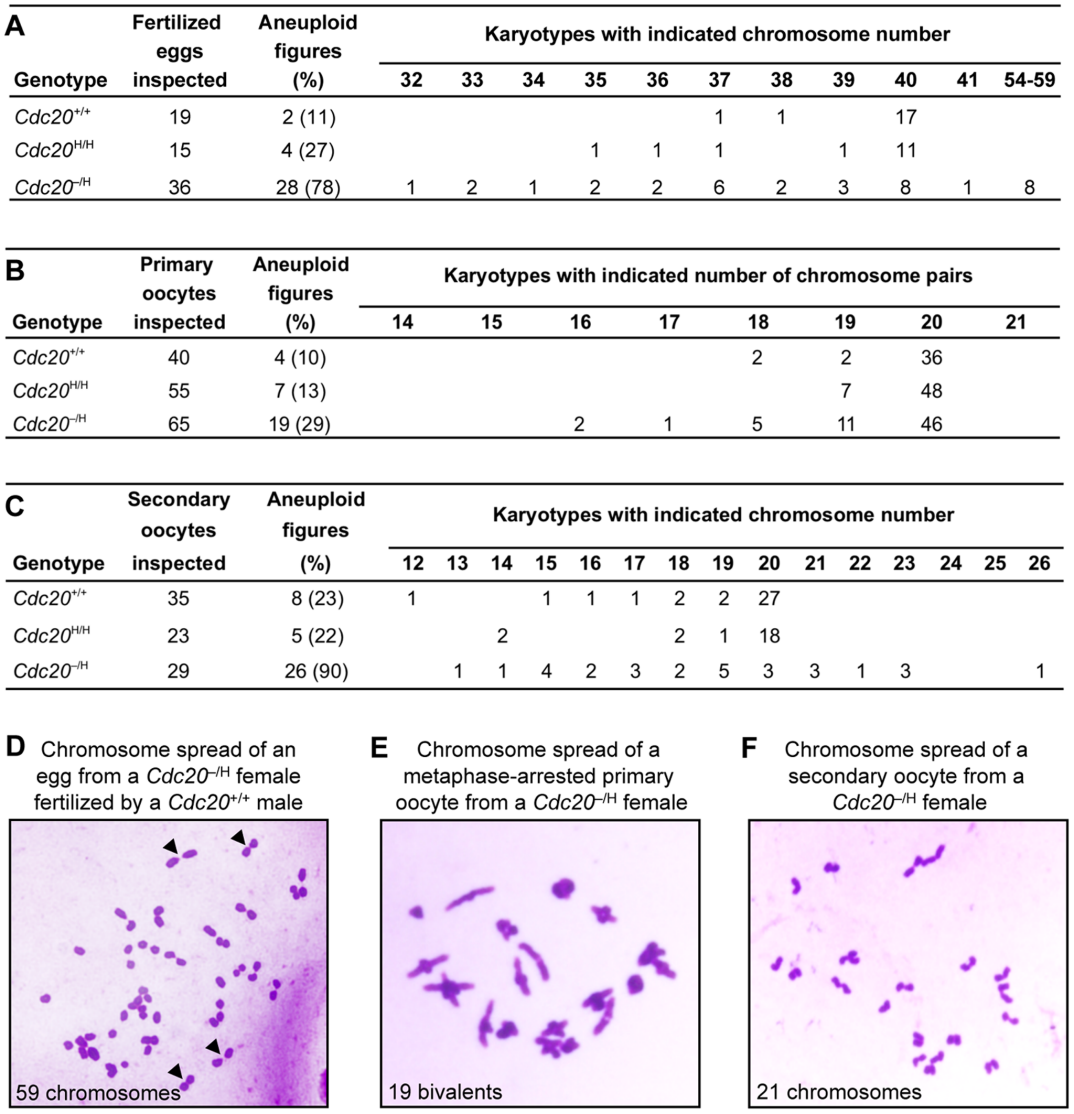
Oocytes and embryos from *Cdc20* ^−/H^ females have abnormal chromosome numbers. (A) Fertilized eggs from *Cdc20* ^−/H^ females x *Cdc20* ^+/+^ males show near-diploid or near-triploid aneuploidy. (B) Mitotic divisions that establish oogonia during fetal development are aneuploidy prone if Cdc20 levels are low. (C) Meiosis I is a prominent source of aneuploidy in *Cdc20* ^−/H^ females. (D) Image of a chromosome spread of a fertilized egg with probable meiosis II failure. Arrowheads mark examples of duplicated chromosomes (most likely oocyte derived). (E) Image of an aneuploid metaphase I of a *Cdc20* ^−/H^ primary oocyte. (F) Image of an aneuploid metaphase II oocyte from a *Cdc20* ^−/H^ female.

We hypothesized that Cdc20 hypomorphism promotes chromosome missegregation during oogenesis, resulting in production of aneuploid embryos that fail to thrive. To test this idea, we collected one-cell stage embryos from *Cdc20* ^+/+^, *Cdc20* ^H/H^ and *Cdc20* ^−/H^ females mated with *Cdc20* ^+/+^ males and prepared metaphase spreads for chromosome counts. We found that 11% of embryos from *Cdc20* ^+/+^ females were aneuploid compared to 27% and 78% of embryos from *Cdc20* ^H/H^ and *Cdc20* ^−/H^ females, respectively (Figure 4A). Aneuploidy was strongly biased toward loss of chromosomes, irrespective of *Cdc20* genotype. Importantly, nearly 30% of aneuploid embryos from *Cdc20* ^−/H^ females had 14 to 19 extra chromosomes (Figure 4A and 4D). We noted that these embryos contained a very high proportion of chromosome pairs (Figure 4D), which suggested that they originated from mature oocytes that had failed to complete meiosis II after fertilization.

Next, we determined whether Cdc20 hypomorphism also leads to erroneous chromosome segregation at earlier stages of oogenesis. During embryogenesis, primordial germ cells migrate to the developing gonad to form oogonia, which expand in number through a series of mitotic divisions before differentiating into primary oocytes that arrest in prophase of meiosis I. To determine whether the early mitotic divisions might contribute to the aneuploidy seen in fertilized eggs, primary oocytes were harvested from ovaries of *Cdc20* ^+/+^, *Cdc20* ^H/H^ and *Cdc20* ^−/H^ females. In mice, primary oocytes normally have 20 paired chromosomes, called bivalents. Primary oocytes from *Cdc20* ^+/+^ and *Cdc20* ^H/H^ females had abnormal numbers of bivalents in 10% and 13% of spreads, respectively (Figure 4B). In contrast, primary oocytes from *Cdc20* ^−/H^ females had considerably more aneuploidy, with 29% of spreads showing abnormal numbers of bivalents (Figure 4B and 4E). These spreads showed no evidence of precocious separation of bivalents, indicating that formation of chiasmata was intact at low Cdc20 levels.

Although Cdc20 insufficiency causes aneuploidy during the early mitotic divisions of oogenesis, aneuploidy rates of primary oocytes were substantially lower than those of fertilized eggs. To explore whether additional aneuploidy occurred during meiosis I, we prepared metaphase spreads from secondary oocytes of *Cdc20* ^+/+^, *Cdc20* ^H/H^ and *Cdc20* ^−/H^ females and counted chromosomes. We found that aneuploidy rates of secondary oocytes from *Cdc20* ^+/+^ and *Cdc20* ^H/H^ females increased modestly to 23% and 22%, respectively (Figure 4C). This verified that the level of Cdc20 protein in oocytes from Cdc20^H/H^ females was enough to let the chromosomes separate correctly at meiosis I. In contrast, a much more dramatic increase was recorded for secondary oocytes from *Cdc20* ^−/H^ females, with 90% of spreads showing numerical chromosome abnormalities (Figure 4C and 4F).

**Figure 5:**
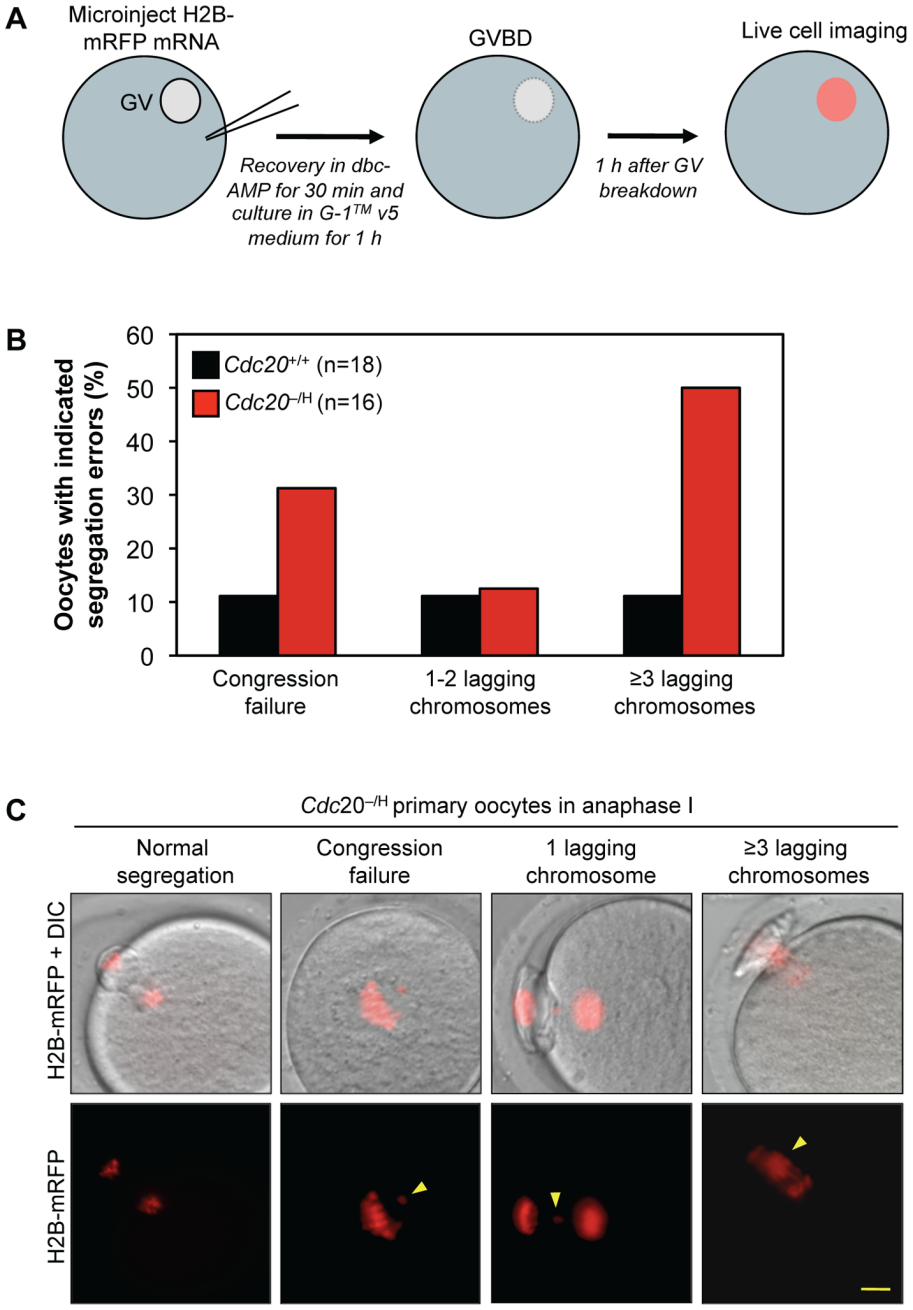
*Cdc20* ^−/H^ oocytes show increased chromosome missegregation in meiosis I. (A) Schematic overview of the experimental procedure. A small amount of H2B-mRFP mRNA was injected into GV-positive *Cdc20* ^+/+^ and *Cdc20* ^−/H^ primary oocytes. After short recovery, oocytes were released from prophase I arrest by removal of dbcAMP. About 1 h after GVBD, we started to monitor chromosome movements by live cell imaging. We note that GVBD itself was not affected by Cdc20 hypomorphism. (B) Percentage *Cdc20* ^+/+^ and *Cdc20* ^−/H^ primary oocytes with the indicated chromosome segregation errors. (C) Examples of *Cdc20* ^−/H^ oocytes undergoing normal or aberrant anaphase I. Arrowheads highlight misaligned and lagging chromosomes. We note that most oocytes with lagging chromosomes were able to complete meiosis I. Bar is 10 µm.

To obtain direct evidence for chromosome missegregation during the first meiotic division of Cdc20 insufficient oocytes, we monitored chromosome movements of *Cdc20* ^+/+^ and *Cdc20* ^−/H^ primary oocytes during meiosis I using time-lapse fluorescence imaging (Figure 5A). To visualize chromosomes we injected in vitro transcribed H2B-mRFP mRNA into the oocytes. In this setup, oocytes from *Cdc20* ^−/H^ females displayed much higher rates of chromosome missegregation than oocytes from *Cdc20* ^+/+^ females (Figure 5B). The two types of errors that were observed are congression failure and chromosome lagging, of which the latter defect was clearly most frequent. Particularly, chromosome lagging incidents involving three or more lagging chromosomes occurred at much higher rates in *Cdc20* ^−/H^ oocytes (Figure 5B and 5C, and Video S1 and Video S2). Thus, consistent with our chromosome counts on secondary oocytes, chromosome segregation errors during meiosis I contribute considerably to the infertility phenotype of *Cdc20* ^−/H^ females.

### Cdc20 Hypomorphism Prolongs Metaphase I

**Figure 6:**
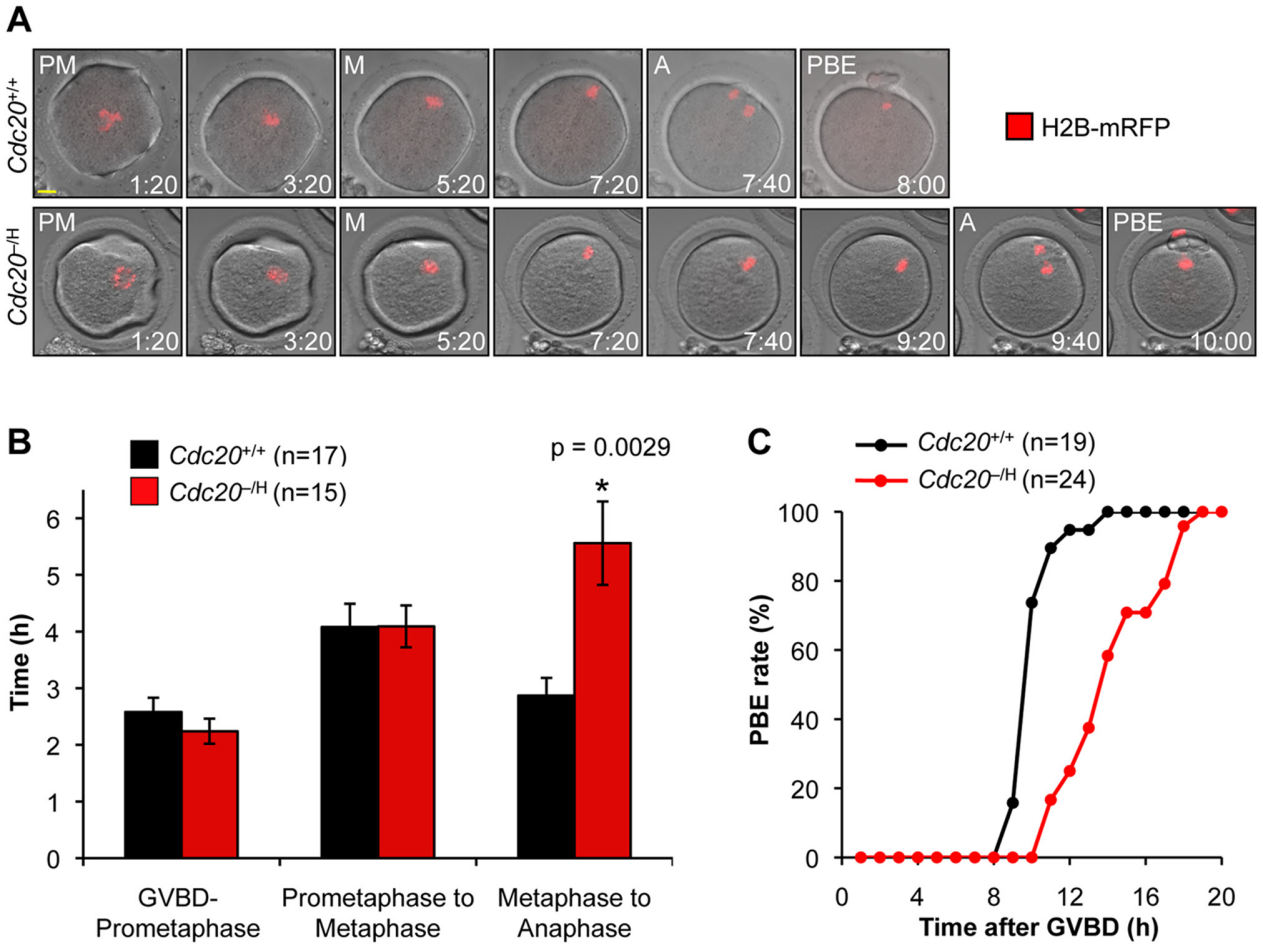
Metaphase I is retarded in *Cdc20* ^−/H^ oocytes. (A) The progression through meiosis I of *Cdc20* ^+/+^ and *Cdc20* ^−/H^ oocytes expressing H2B-mRFP was monitored by live-cell microscopy and typical examples of image sequences are shown. Time after GVBD is indicated in each image (h:min). Abbreviations: PM, prometaphase; M, metaphase; A, anaphase; and PBE, polar body extrusion. Scale bar is 10 µm. (B) Measurement of the timing of meiosis I of H2B-mRFP-expressing *Cdc20* ^+/+^ and *Cdc20* ^−/H^ oocytes by live-cell imaging. Oocytes with congression defects were excluded from the experiment. Data shown are mean ± SEM. *p<0.05 (student t-test). (C) Polar body extrusion rates of cultured *Cdc20* ^+/+^ and *Cdc20* ^−/H^ primary oocytes as assessed by time-lapse microscopy (DIC imaging). The time at which 50% of oocytes had completed PBE was 9.5 h for *Cdc20* ^+/+^ oocytes and 13.6 h for *Cdc20* ^−/H^ oocytes.

Orderly progression of oocytes through meiosis I is controlled by the APC/C, which prompted us to examine whether timing of meiosis I is deregulated at low Cdc20 levels. *Cdc20* ^+/+^ and *Cdc20* ^−/H^ oocytes were injected with H2B-mRFP mRNA and observed by time-lapse microscopy while executing meiosis I. We found that the time from germinal vesicle breakdown (GVBD) to metaphase was similar in *Cdc20* ^+/+^ and *Cdc20* ^−/H^ oocytes (Figure 6A and 6B), which is consistent with the notion that Cdh1 functions as the primary ACP/C activator during the early stages of meiosis I [12]. However, the average time from metaphase entry to anaphase onset was about two times longer in *Cdc20* ^−/H^ oocytes than in *Cdc20* ^+/+^ oocytes (Figure 6A and 6B). This delay was unlikely to be due to chromosome segregation errors as oocytes with misaligned or lagging chromosomes were excluded from the analysis. Consistent with delayed metaphase progression, PBE extrusion was markedly delayed in *Cdc20* ^−/H^ oocytes (Figure 6C). Taken together, these data indicate that the timing of metaphase I is subject to deregulation when the amount of Cdc20 protein is limited.

### Low Cdc20 Impairs Degradation of Mitotic Cyclins and Securin in Metaphase I

**Figure 7:**
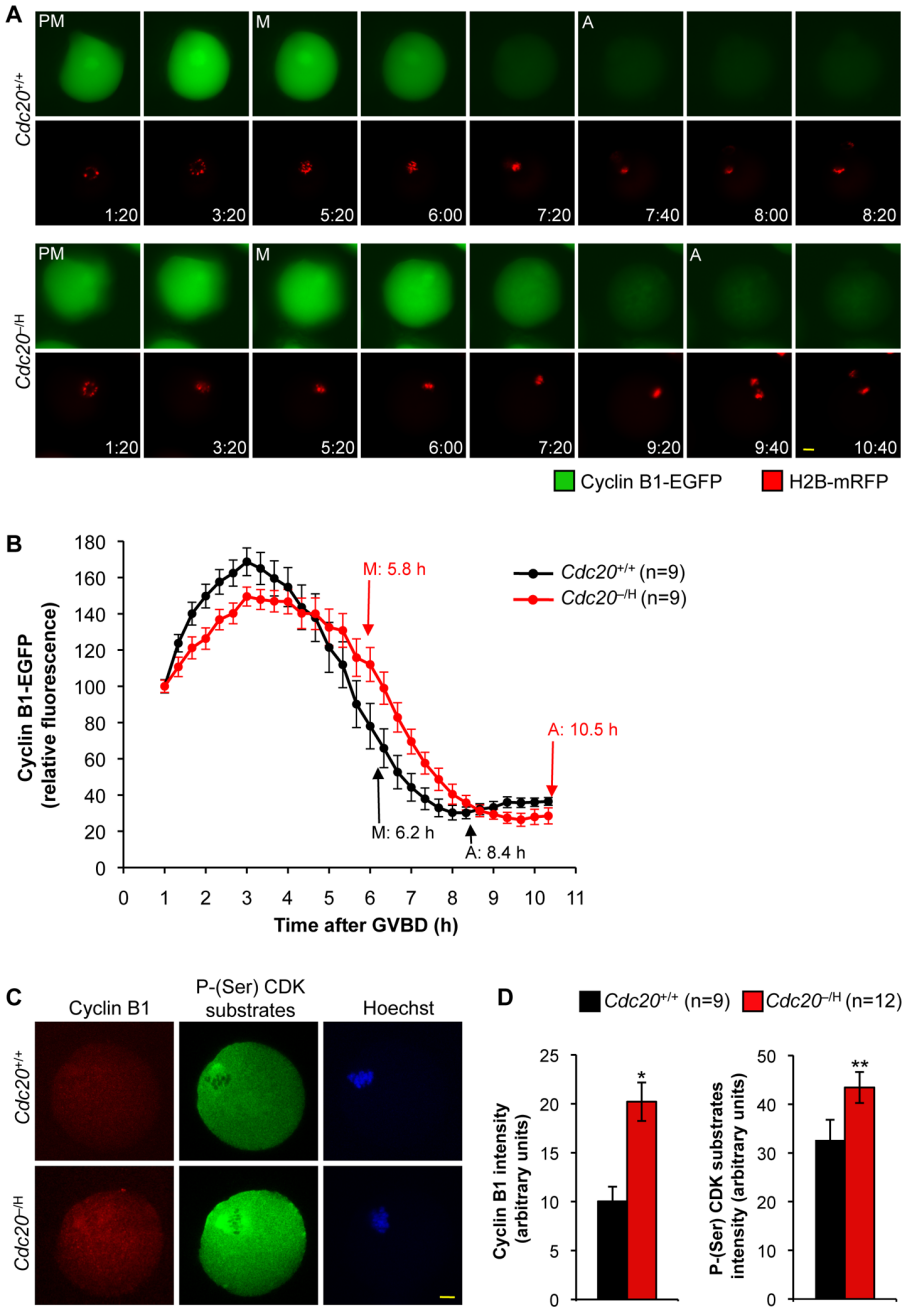
Cyclin B1 degradation is delayed during metaphase I if Cdc20 are low. (A,B) Kinetics of cyclin B1-EGFP degradation during meiosis I. *Cdc20* ^+/+^ and *Cdc20* ^−/H^ primary oocytes were collected and injected with transcripts encoding cyclin B1-EGFP and H2B-mRFP prior to GVBD. Cyclin B1-EGFP degradation was monitored by time lapse microscopy as oocytes progressed through meiosis I. (A) Still images illustrating that cyclin B1 degradation is delayed in *Cdc20* ^−/H^ primary oocytes. Time after GVBD (h:min) is indicated for each image. Scale bar is 10 µm. (B) Graph showing the mean cyclin B1-EGFP fluorescence intensities of the indicated numbers of *Cdc20* ^+/+^ and *Cdc20* ^−/H^ oocytes. For each oocyte, the fluorescence intensity was normalized to the intensity recorded 1 h after GVBD. Abbreviations in (A,B) are as in Figure 6A. Error bars represent SEM. (C) Metaphase I oocytes of the indicated genotypes stained for cyclin B1, p-(Ser) Cdk substrates, and DNA (Hoechst). Bar = 10 µm. Note that signals of both cyclin B1 and p-(Ser) Cdk substrates are increased in the *Cdc20* ^−/H^ oocyte. (D) Quantification of cyclin B1 and p-(Ser) Cdk substrate signals. *p = 0.001 versus *Cdc20* ^−/H^ metaphase (unpaired t test). **p = 0.033 versus *Cdc20* ^−/H^ metaphase (unpaired t test). Error bars represent SEM.

To explore the mechanism underlying the chromosome missegregation phenotype of *Cdc20* ^−/H^ primary oocytes, we measured the rate of degradation of two key APC/C^Cdc20^ substrates, cyclin B1 and securin [12]. In the first set of experiments, we injected *Cdc20* ^−/H^ and *Cdc20* ^+/+^ primary oocytes with mRNA encoding cyclin B1-EGFP and monitored the degradation of fluorescent protein by live-cell imaging. Oocytes were coinjected with H2B-mRFP mRNA to accurately assess the timing of cyclin B1-EGFP degradation. As illustrated in Figure 7A and 7B, *Cdc20* ^+/+^ oocytes degraded most of their cyclin B1-EGFP during late prometaphase and early metaphase. *Cdc20* ^−/H^ oocytes entered metaphase I around the same time as *Cdc20* ^+/+^ oocytes. However, they did so with relatively high cyclin B1-EGFP protein levels and completed substrate degradation ∼2 h later than *Cdc20* ^+/+^ oocytes. To confirm that cyclin B1 degradation was delayed, we used indirect immunofluorescence to measure endogenously expressed cyclin B1 levels of *Cdc20* ^+/+^ and *Cdc20* ^−/H^ oocytes in metaphase I. As shown in Figure 7C and 7D, cyclin B1 levels were indeed higher in *Cdc20* ^−/H^ oocytes than in *Cdc20* ^+/+^ oocytes. Importantly, these oocytes also showed elevated levels of phosphorylated Cdk substrates (Figure 7C and 7D), suggesting that the rise in cyclin B1 expression resulted in increased cyclin B1-Cdk1 activity in metaphase I.

**Figure 8:**
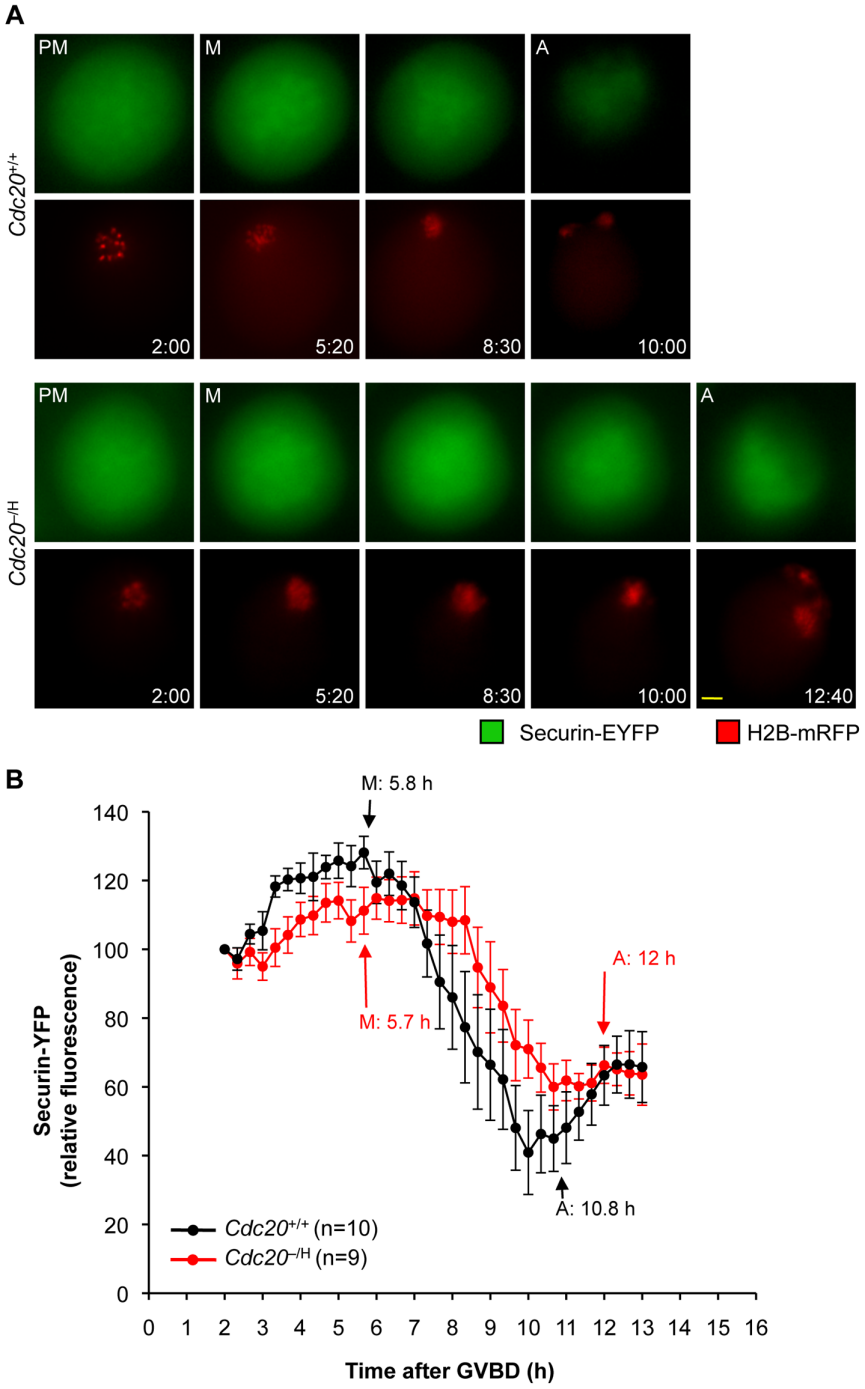
*Cdc20* ^−/H^ oocytes have impaired securin destruction in metaphase I. Rates of securin-EYFP degradation during meiosis I. *Cdc20* ^+/+^ and *Cdc20* ^−/H^ primary oocytes were injected with transcripts encoding H2B-mRFP and securin-YFP. After induction of GVBD, degradation of securin-EYFP was followed by time-lapse microscopy. (A) Time-lapse microscopy images illustrating that securin-EYFP degradation is delayed in *Cdc20* ^−/H^ primary oocytes. The time after GVBD (h:min) is indicated. Scale bar represents 10 µm. (B) Graph showing the mean securin-EYFP fluorescence intensities for *Cdc20* ^+/+^ and *Cdc20* ^−/H^ primary oocytes. The fluorescence intensity for each oocyte was normalized to the intensity recorded 2 h after GVBD. Abbreviations in (A,B) are as in Figure 6A. Error bars represent SEM.

**Figure 9:**
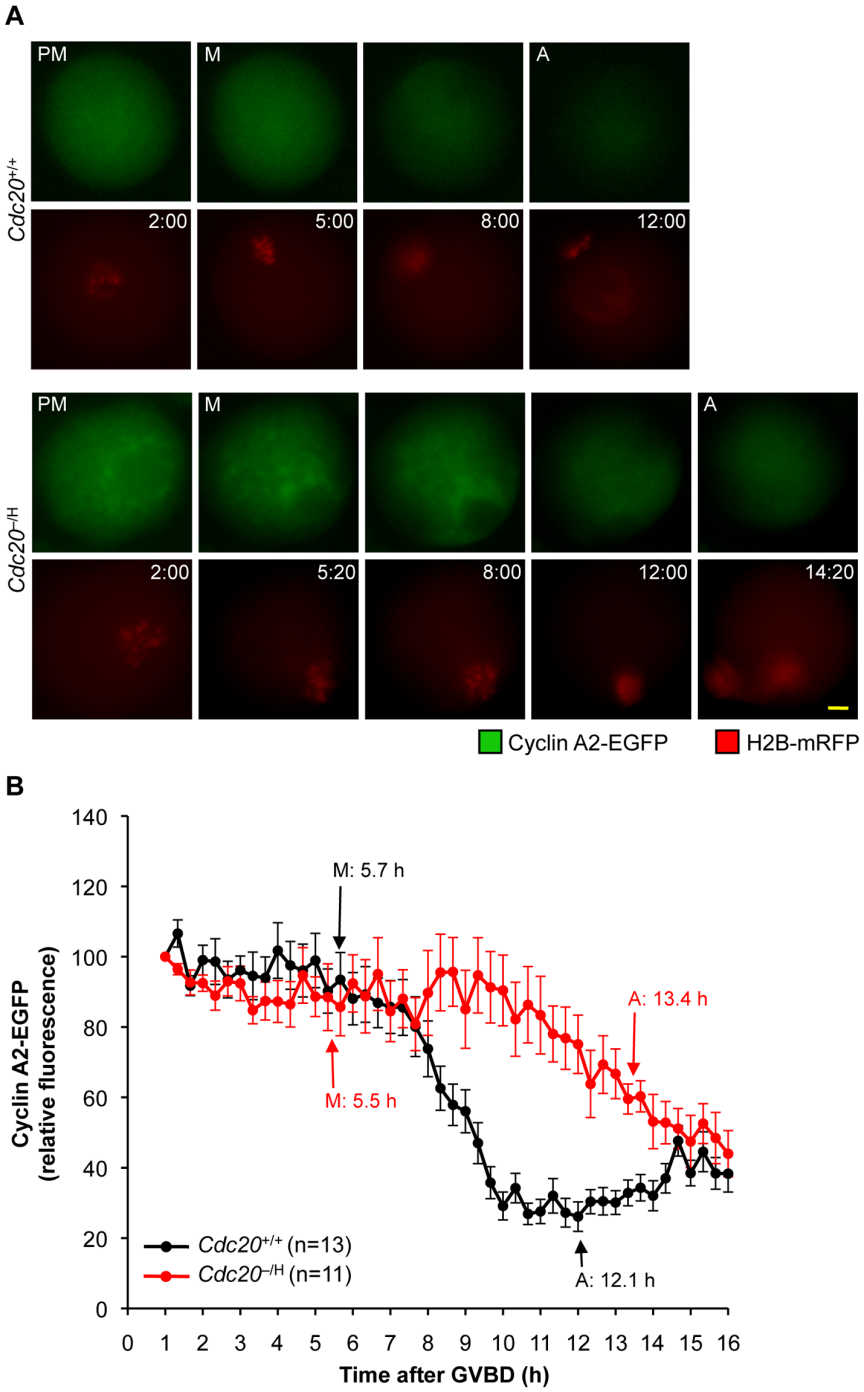
*Cdc20* ^−/H^ oocytes show inefficient cyclin A2 destruction in metaphase I. (A) Still images illustrating that cyclin A2-EGFP degradation is impaired in *Cdc20* ^−/H^ primary oocytes (the time after GVBD is indicated). Scale bar represents 10 µm. (B) Graph showing the mean cyclin A2-EYFP fluorescence intensities for *Cdc20* ^+/+^ and *Cdc20* ^−/H^ primary oocytes. The fluorescence intensity for each oocyte was normalized to the intensity recorded 1 h after GVBD. Error bars represent SEM.

Next, we coinjected securin-EYFP [24] and H2B-mRFP mRNA into *Cdc20* ^−/H^ and *Cdc20* ^+/+^ primary oocytes. We noticed that expression of securin-EYFP protein markedly inhibited PBE even in *Cdc20* ^+/+^ oocytes (data not shown), but were able to control this problem by reducing the concentration of the injected securin-EYFP mRNA. In *Cdc20* ^+/+^ oocytes, onset of securin-EYFP degradation typically coincided with metaphase entry and then rapidly progressed until anaphase onset (Figure 8). In *Cdc20* ^−/H^ oocytes, however, securin-EYFP protein degradation did not start until mid metaphase. Degradation not only started later, but was also less efficient, resulting in anaphase entry with higher than normal levels of securin-EYFP. In a recent study, McGuinness et al. demonstrated that the timing of cyclin A2 degradation in primary oocytes is similar to that of securin [17], which is surprising given that mitotic cells fully degrade this cyclin in prometaphase. In light of these findings, we wanted to examine whether the degradation of cyclin A2 was impaired in *Cdc20* ^−/H^ oocytes. As for securin-EYFP, cyclin A2-EGFP inhibited PBE in *Cdc20* ^+/+^ oocytes when expressed at high levels (data not shown), but again we were able to control this problem by injecting low amounts of transcript. Consistent with the earlier data [17], *Cdc20* ^+/+^ primary oocytes rapidly destroyed cyclin A2-EGFP in metaphase I (Figure 9). In contrast, both the onset and the rate of cyclin A2-EGFP were substantially reduced in *Cdc20* ^−/H^ oocytes. Strikingly, *Cdc20* ^−/H^ oocytes again entered anaphase I with higher substrate levels than *Cdc20* ^+/+^ oocytes. Taken together, the above data demonstrate that multiple APC/C substrates are inefficiently degraded when Cdc20 levels are low, raising the possibility that persistent cyclin-CDK activity in metaphase I might underlie, at least in part, the chromosome missegregation phenotype of Cdc20^−/H^ oocytes.

It is conceivable that delayed cyclin and securin degradation impairs separase activation, and therefore proper cleavage of cohesin along chromosome arms of bivalents prior to anaphase onset. To test for this possibility, we collected *Cdc20* ^−/H^ and *Cdc20* ^+/+^ primary oocytes, cultured them in vitro until they arrested in metaphase II and then stained chromosomes for the presence of Rec8, a meiosis specific component of the cohesin complex [25], [26]. While Rec8 staining was readily detectable along chromosome arms of metaphase I chromosomes, no such staining was detectable in metaphase II oocytes, irrespective of *Cdc20* genotype (Figure S1), implying that *Cdc20* ^−/H^ oocytes generated sufficient separase activity for complete cleavage of Rec8. Furthermore, core mitotic checkpoint proteins that are involved in kinetochore assembly, kinetochore-microtubule and/or spindle assembly checkpoint activation, such as Bub1, BubR1, and Mad2, were normally localized at kinetochores of *Cdc20* ^−/H^ primary oocytes (Figure S2).

### Aneuploidy Rates during Male Meiosis I Are Relatively Low

**Figure 10:**
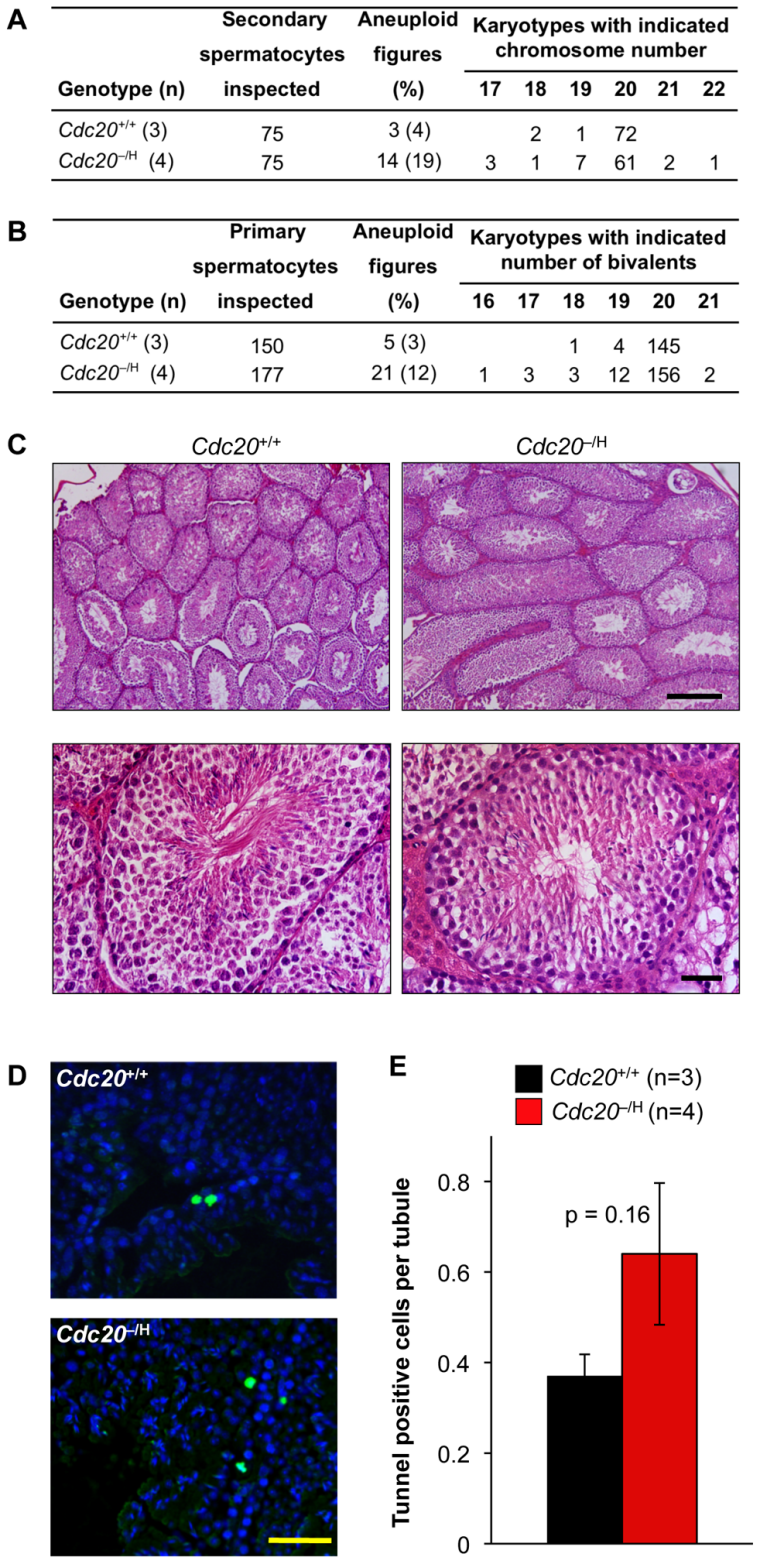
Spermatogenesis is aneuploidy prone at low Cdc20 levels. (A) *Cdc20* ^−/H^ males are much less prone to aneuploidy in meiosis I than *Cdc20* ^−/H^ females. (B) Mitotic divisions that amplify spermatogonia appear to be prone to aneuploidy when Cdc20 levels are low. (C) H/E-stained testis sections from *Cdc20* ^+/+^ and *Cdc20* ^−/H^ males. Paraffin sections (5 µm) were from 5 month-old mice. Bar in top and bottom images represent 200 µm, and 25 µm, respectively. (D) Representative images of TUNEL-stained testis sections from *Cdc20* ^+/+^ and *Cdc20* ^−/H^ males. TUNEL-positive cells are green. Cell nuclei were visualized by Hoechst staining. Scale bar represents 100 µm. (E) Quantitation of apoptosis in testes from *Cdc20* ^+/+^ and *Cdc20* ^−/H^ males. The number of TUNEL-positive cells was counted in 50 tubules. Only tubules that were cross-sectioned were considered. There is no statistical difference between both groups (student t-test).

*Cdc20* ^−/H^ males appeared to have normal fertility, predicting that male meiosis I is much less sensitive to Cdc20 hypomorphism. To verify this, we prepared chromosome spreads of testicular cell suspensions from *Cdc20* ^+/+^ and *Cdc20* ^−/H^ mice and performed chromosome counts on secondary spermatocytes. Although aneuploidy was 5-fold higher at low than at normal Cdc20 levels (Figure 10A), secondary spermatocytes of *Cdc20* ^−/H^ males had much lower aneuploidy rates than secondary oocytes of *Cdc20* ^−/H^ females (19% versus 90%). Chromosome counts on primary spermatocytes revealed a 4-fold increase in aneuploidy due to Cdc20 hypomorphism, with 12% of spreads showing abnormal numbers of bivalents (Figure 10B), suggesting that the mitotic divisions that spermatogonia have to undergo to produce primary spermatocytes are error prone at low Cdc20 levels. The rather modest increase in aneuploidy from 12% to 19% as primary spermatocytes develop into secondary spermatocytes underscores that the fidelity of male meiosis I remains quite high at low Cdc20 levels. Furthermore, histology and apoptosis rates were normal in testis of *Cdc20* ^−/H^ males, as judged by hematoxylin and eosin (H/E) and TUNEL staining of testis sections, respectively (Figure 10C–10E).

## Discussion

By generating a series of mice with graded reduction in Cdc20 levels, we discovered a remarkably sharp threshold for Cdc20 expression in female germ cells below which chromosome segregation errors occur at high frequency, leading to production of aneuploid eggs that are fertilization competent but fail to progress beyond the first few embryonic divisions. On the other hand, low Cdc20 levels are well tolerated by somatic tissues and have no overt impact on the overall health and life expectancy of mice. These findings raise the intriguing possibility that hypomorphic *Cdc20* alleles may be responsible for unexplained fertility problems in otherwise healthy women.

Because aneuploidy has been associated with reduced cell growth and survival [27], [28], one might have predicted that oogenesis would be severely disrupted in Cdc20 hypomorphic mice. Surprisingly, however, we did not observe significant alterations in the number and morphology of follicles and corpora lutea in these mice. These findings suggest that cellular pathways that might inhibit cell proliferation or induce cell death in response to chromosome missegregation are either not active in female germ cells or require a higher threshold for activation than in somatic cells [29]. Our finding that folliculogenesis was unperturbed was also unexpected in light of studies showing that depletion of Cdc20 from primary oocytes by a morpholino causes metaphase I arrest [12]. For somatic cells it has been estimated that metaphase arrest requires a 20-fold or higher reduction in cellular Cdc20 levels [30]. We suspect that morpholino treatment reaches this level of reduction, whereas Cdc20 hypomorphism does not.

In systematically karyotyping primary and secondary oocytes and fertilized eggs, we discovered that Cdc20 hypomorphism promotes aneuploidization at different stages of oogenesis, involving both mitotic and meiotic divisions. The highest increase in aneuploidy, however, occurred in the first meiotic division. The most prominent segregation errors that we observed during meiosis I are chromosome misalignment and chromosome lagging. Previous studies in HeLa and Ptk1 cells uncovered that cyclin A2 overexpression causes chromosome misalignment [31], suggesting that alignment defects in Cdc20 hypomorphic oocytes might be related to their inability to destroy cyclin A2 in a timely fashion. Resolution of chiasmata requires removal of cohesin from chromosome arms, which involves cleavage of the cohesin subunit Rec8 by separase [32]. In turn, activation of separase requires APC/C-mediated degradation of securin and cyclin B, both of which are delayed in Cdc20 hypomorphic primary oocytes. Thus, it is possible that Cdc20 hypomorphic oocytes do not have enough APC/C activity to fully activate separase and properly resolve chiasmata, thereby prompting chromosome lagging and aneuploidization. Arguing against this explanation is the fact that chromosome spreads of Cdc20 hypomorphic metaphase II oocytes did not contain any bivalents or chromosomes that stained positive for Rec8 along chromosome arms. Alternatively, chromosome lagging in Cdc20 hypomorphic oocytes might be caused by microtubule-kinetochore attachment defects [33], [34]. For instance, delayed degradation of cyclin B1 (or other APC/C substrates) might promote such defects by disrupting key components of the mechanisms that establish syntelic attachment or that correct merotelic or amphitelic attachments. We found that two mitotic checkpoint proteins required for proper microtubule-chromosome attachment, Bub1 and BubR1, were normally localized at kinetochores of Cdc20 hypomorphic oocytes. However, it should be emphasized that microtubule-kinetochore attachment is a complex process requiring many different proteins, any of which could be deregulated in our mutant oocytes. Interestingly, in somatic cells, a small fraction of Cdc20 accumulates at kinetochores during mitosis [35], which raises the possibility that Cdc20 might have a more direct role in establishing proper microtubule-kinetochore attachments.

Our finding that a significant percentage of *Cdc20* ^−/H^ primary oocytes were already aneuploid before resuming meiosis I suggests that the mitotic divisions by which primordial germ cells develop into primary oocytes are prone to chromosome missegregation when Cdc20 levels are below a certain threshold. Due to technical limitations, it was not possible to verify this experimentally. The precise impact of Cdc20 hypomorphism on meiosis II is difficult to decipher, largely because nearly all oocytes are aneuploid after meiosis I. However, the presence of a sizeable amount of near triploid fertilized eggs strongly suggests that Cdc20 insufficiency can cause failure of maternal sister chromatids to separate during meiosis II, although we note that it cannot be excluded that the preexisting aneuploidy rather than the low Cdc20 levels drive the separation defect. It could be argued that embryos from *Cdc20* ^−/H^ females bred to *Cdc20* ^+/+^ males might fail to thrive due to a potential lack of Cdh1 expression in the early embryos, rendering embryonic mitotic divisions particularly dependent on Cdc20. However, this explanation seems unlikely because *Cdh1* has been shown to be expressed in two-cell stage mouse embryos [23]. Furthermore, it should be considered that the embryos from *Cdc20* ^−/H^ females bred to *Cdc20* ^+/+^ males that fail to thrive had either *Cdc20* ^+/−^ or *Cdc20* ^+/H^ genotypes. Embryos of both these genotypes show normal survival rates when derived from *Cdc20* ^+/−^ and Cdc20^+/H^ females crossed with *Cdc20* ^+/+^ males (Figure 2B and 2C), further supporting the idea that aneuploidy acquired during oogenesis is largely responsible for the early death of embryos from *Cdc20* ^−/H^ females.

An intriguing finding was that the fertility problems of *Cdc20* ^−/H^ mice are restricted to females, even though our analysis of aneuploidy in primary and secondary spermatocytes demonstrated that mitotic and meiotic divisions of male germ cells are prone to aneuploidy. However, the key difference between males and females that probably accounts for their distinct fertilities is that the rate of aneuploidization during meiosis I is substantially higher in females than in males. Why might female meiosis I be more sensitive to Cdc20 hypomorphism? A recent study of mouse oocytes suggests that mammals have a unique mechanism for control of meiosis I in that they require APC/C^Cdh1^ activity for progression through prometaphase I [12]. Cdc20 is targeted for destruction by this early APC/C activity and needs to be re-synthesized during metaphase I to enable anaphase onset. It is possible that Cdc20 destruction in prometaphase I only occurs in females, perhaps creating a higher degree of Cdc20 insufficiency in oocytes than in spermatocytes.

## Materials and Methods

### Generation of Cdc20 Mutant Mice

The gene targeting procedure used to produce the hypomorphic *Cdc20* allele (H) was as previously described [22]. To generate the targeting construct, *Cdc20* gene fragments of 3.9 kb (spanning exons 1–3) and 4.7 kb (spanning exons 4–10) were PCR amplified from 129Sv/E genomic DNA and cloned into HindIII-XbaI and SalI-NotI sites of pNTKV1901 (Stratagene). The targeting construct was linearized with NotI and electroporated into TL1 129Sv/E ES cells. Transfectants were selected in 350 µg/ml G418 and 0.2 µM FIAU, and expanded for Southern blot analysis using a 710 bp 3′ external probe on EcoR1-digested genomic ES cell DNA. This probe was amplified by PCR from 129Sv/E genomic DNA using the following primers: 5′-CATGGCTGGTTTGGGAGAGAATGC TG-3′ and 5′-CACAACACAGTTCATCTTCCCAGTG-3′. Chimeric mice were produced by microinjection of targeted ES cell clones with 40 chromosomes into C57BL/6 blastocysts. Chimeric males were mated with C57BL/6 females and germline transmission of the *Cdc20* ^H^ allele was verified by PCR analysis of tail DNA from pups with a agouti coat color. The *Cdc20* ^−^ allele used in our studies was derived from gene trap ES clone XE368 (purchased from BayGenomics). The following primer combinations were used for PCR genotyping of mice used in our studies: primers a (5′-CAGAAAGCCTGGTCTCTCAACCTG-3′) and b (5′-CACAGTAGTCATTCCGGATT TCGGG-3′) for *Cdc20* ^+^; primers b and c (5′-TCCATTGCTCAGCGGTGCTG -3′) for *Cdc20* ^H^; and primers d (5′-GTATCCAACCATGGCCAAGGTGGCTGAG-3′) and e (5′-TATACGAAGTTATCGATCTGCGATCTGC-3′) for *Cdc20* ^−^. All mouse experiments were conducted after approval of the Mayo Clinic Committee on Animal Care and Use. All mice in the study were of a 129Sv/E x C57BL/6 mixed genetic background.

### Fertility Analysis and Histology

Female fertility was measured by breeding 2-month-old females of various *Cdc20* genotypes to 2- to 3-month-old wild-type males for a 3-month period. During this period, we recorded, for each female, the number of vaginal plugs (to determine whether females showed normal mating behavior), the number of litters produced, and the amount of pups delivered. Histological evaluation of testes and ovaries were as previously described [11]. Follicles and corpora lutea were counted in five ovary sections of each mouse. Follicle classification was according to Pedersen and Peters [36]. TUNEL staining was done on 5 µm testis sections using an in situ cell death detection kit from Roche.

### Western Blot Analysis and Indirect Immunofluorescence

Western blot analysis was performed as described earlier [37]. Extracts of MEFs, splenocytes, and bone marrow were prepared in PBS containing 0.1% NP40, 10% glycerol and complete protease inhibitor cocktail (Roche). Extracts were centrifuged at 20,000 *g* for 15 min (4°C), and supernatants collected for electrophoresis. Quantitation of relative Cdc20 protein levels in *Cdc20* ^+/H^, *Cdc20* ^+/−^, *Cdc20* ^H/H^ and *Cdc20* ^−/H^ testis and ovary, and *Cdc20* ^−/H^ MEFs, spleen, and bone marrow was done as previously described [38]. Briefly, Cdc20 western blot signals obtained with rabbit Cdc20 antibody from Santa Cruz (SC-8358), were quantified using ImageJ software (http://rsbweb.nih.gov) and normalized to background and β-actin (Sigma A5441) or α-tubulin (Sigma, T-9026) signals. Values obtained were normalized to those of corresponding wild-type tissues and MEFs, where wild-type signals were set at 100. Normalized signal values were converted to percent protein using the graph of Figure S3. Relative Cdc20 protein amounts represent the average of at least two independent samples.

Indirect immunofluorescence was performed as previously described [37], [39]. Immunofluorescence images were captured using a Carl Zeiss LSM 510 laser-scanning microscope with a c-Apochromat 100× oil immersion objective. Fluorescent signals from cyclin B1 and P-(Ser) CDKs substrate labelings were quantitated using ImageJ software. The mean fluorescence intensity was determined after background subtraction of images transformed to 8 bits grayscale. The following primary antibodies were used: cyclin B1 (Calbiochem, PC-133), P-(Ser) CDKs substrate (Cell Signaling, #2324), BubR1(1-350) [11], human anti-centromere antibody (Antibodies Inc, 15-235-0001), Bub1(25-165) [28], Mad2 (polyclonal anti-mouse full-length Mad2 antibodies generated in a rabbit), and Rec8 (kindly provided by Dr. J. Lee [25]).

### Isolation and Culture of Oocytes and Fertilized Eggs

Primary oocytes were isolated from ovaries of 3- to 4-week-old *Cdc20* ^+/+^ and *Cdc20* ^−/H^ mice as described [40], and cultured in micro-drops of G-1 v5 plus medium (Vitrolife) under embryo-tested paraffin oil (Vitrolife). In case primary oocytes were used in mRNA microinjection experiments, 50 µg/ml dibutyryl cyclic AMP (dbcAMP) was added to the G-1 v5 plus medium to inhibit GVBD. To obtain secondary oocytes, 3- to 4-week-old *Cdc20* ^+/+^ and *Cdc20* ^−/H^ females were injected with pregnant mare serum gonadotropin (PMSG; 5 IU/mouse, Sigma G4527) and 46 h later with human chorionic gonadotropin (hCG; 5 IU/mouse, Sigma C0684). Eighteen h after the hCG injection, ovaries were collected and secondary oocytes harvested from oviducts. Metaphase II-arrested oocytes for Rec8 immunostaining experiments were prepared by culturing primary oocytes from ovaries of 3- to 4-week-old *Cdc20* ^+/+^ and *Cdc20* ^−/H^ mice in G-1 v5 plus medium until they arrested in metaphase. Fertilized eggs were produced by mating 6- to 12 week-old *Cdc20* ^+/+^ and *Cdc20* ^−/H^ females with *Cdc20* ^+/+^ males. The next morning, one-cell stage embryos were harvested from oviducts and freed of cumulus cells as described [41]. Embryo culturing was done in micro-drops of G-1 v5 plus medium as described [42]. Embryos were photographed daily from day E0.5 to E4.5.

### Chromosome Counts on Oocytes, One-Cell Stage Embryos, and Spermatocytes

For chromosome counts on oocytes and one-cell stage embryos, the procedure of Tarkowski [43] was followed. Briefly, freshly harvested secondary oocytes and fertilized eggs were cultured for 20 h at 37°C in medium containing 0.5 µg/ml colcemid, incubated in 1% sodium citrate for 20 min at RT and transferred to glass slides. Ethyl alcohol and glacial acetic fixative (3∶1) was dropped on the zygotes and secondary oocytes three times. Air-dried slides were Giemsa stained and chromosomes counted using a light microscope with a 100× objective. Primary oocytes were collected and cultured in micro-drop cultures of G-1 v5 plus medium. Upon GVBD, primary oocytes were harvested and chromosome spreads prepared. For chromosome counts on spermatocytes, testes were collected and minced between two microscope slides. Released cells were suspended in 5 ml PBS, centrifuged at 1,000 rpm for 5 min, resuspended 5 ml 0.075 M KCl, and incubated at RT for 30 min. Cells were fixed in Carnoy's solution, washed, and finally resuspended in 0.5 ml fixative. Twenty-five µl aliquots were dropped onto pre-wetted microscope slides and chromosomes were stained with Giemsa.

### Live-Cell Imaging of Cultured Primary Oocytes

To measure the accuracy of chromosome segregation during meiosis I, chromosome movements of primary oocytes were followed by time-lapse microscopy. To this end, H2B-mRFP mRNA was produced by *in vitro* transcription using the T3 mMESSAGE mMACHINE kit (Ambion Inc). Using a Femtojet microinjector (Eppendorf), GV-stage primary oocytes were microinjected with 5–10 picoliter of mRNA solution containing 0.5 µg/ml H2B-mRFP mRNA [44]. Injected oocytes were allowed to recover for 30 min in micro-drops of M2 medium containing 50 µg/ml dbcAMP and then transferred to 35 mm glass-bottomed culture dishes (MatTek Corporation) containing G-1 v5 plus medium without dbcAMP to induce GVBD. Chromosome movements were followed using a Zeiss Axio Observer Z1 system with CO2 Module S, TempModule S, Heating Unit XL, Pln 40x/0.6 Ph2 DICIII objective, AxioCam MRm camera, and AxioVision 4.6 software [45]. The temperature of the imaging medium was kept at 37°C. Images were collected at interframe intervals of 20 min.

To analyze timing of meiosis I, the time intervals from GVBD to prometaphase, prometaphase to metaphase, and metaphase to anaphase were measured. Importantly, only H2B-mRFP mRNA-injected *Cdc20* ^+/+^ and *Cdc20* ^−/H^ oocytes progressing through meiosis I without any chromosome segregation errors were included in our timing analysis.

To determine polar body extrusion rates, *Cdc20* ^+/+^ and *Cdc20* ^−/H^ oocytes were collected and monitored by differential interference contrast (DIC) time-lapse microscopy as they progressed through meiosis I.

To analyze the degradation kinetics of mitotic cyclins and securin, coding sequences for cyclin B1-EGFP, securin-EYFP and cyclin A2-EGFP were cloned into pBluescript RN3 or pMDL2 [46], and mRNAs were produced by *in vitro* transcription as described above. GV-stage primary oocytes were microinjected with 5–10 picoliter of mRNA solutions containing 0.5 µg/ml H2B-mRFP +0.5 µg/ml cyclin B1-EGFP, 0.1 µg/ml H2B-mRFP +0.1 µg/ml securin-YFP, or 0.1 µg/ml H2B-mRFP +0.1 µg/ml cyclin A2-EGFP. Injected oocytes were allowed to recover for 30 min in micro-drops of M2 medium containing 50 µg/ml dbcAMP and then transferred to 35 mm glass-bottomed culture dishes. Time-lapse microscopy was initiated 1 or 2 h after GVBD to allow for expression of fluorescent protein-tagged APC/C substrates. Images were collected at interframe intervals of 20 min. Quantification of fluorescence levels was as follows. For each oocyte and for each time point, images detecting mRFP, EGFP/EYFP, and DIC were acquired. Time-lapse images were then exported as grayscale “avi” uncompressed files. Videos were opened using ImageJ using avi reader plugin. DIC images were used to highlight the area occupied by the oocyte using the freehand tool in ImageJ. The highlighted area was moved to the corresponding EGFP/EYFP image and the mean fluorescence intensity within this area measured after background subtraction. Mean fluorescence intensities were expressed in arbitrary units. The value of time zero (the fluorescence intensity for the first image acquired) was considered 100% and the subsequent time-lapse intensities were normalized against it. Excel T-TEST software was used for statistical analyses.

## Supporting Information

Figure S1 Chromosome missegregation in *Cdc20* ^-/H^ primary oocytes does not seem to involve non-disjunction of bivalents. (A) A *Cdc20* ^−/H^ primary oocyte in metaphase I stained for the meiotic cohesin component Rec8 [46], centromeres (ACA) and DNA (Hoechst). Note that Rec8 signals are localized along chromosome arms of bivalents. (B) *Cdc20* ^+/+^ and *Cdc20* ^−/H^ primary oocytes were cultured until metaphase II arrest, collected and stained for Rec8, centromeres and DNA. Note that chromosome arms in metaphase II are negative for Rec8 irrespective of genotype, indicating the lack of bivalents. Scale bar represents 10 µm.
(0.61 MB TIF)

Figure S2 Mitotic checkpoint proteins properly localize to kinetochores of *Cdc20* ^−/H^ primary oocytes. *Cdc20* ^+/+^ and *Cdc20* ^−/H^ primary oocytes were harvested from ovaries and cultured until they had progressed to metaphase I (∼7 h after GVBD). Oocytes were fixed and immunostained for centromeres (ACA antibody) and either Bub1, BubR1 or Mad2. DNA was visualized by Hoechst staining. Scale bar represents 10 µm.
(1.22 MB TIF)

Figure S3 Percent Cdc20 protein plotted versus the average band intensity on western blots.
(0.14 MB TIF)

Video S1 Example of a *Cdc20* ^−/H^ oocyte undergoing chromosome missegregation during meiosis I. Chromosomes (red) were marked by injection of H2B-mRFP prior to GVBD. Note the presence of lagging chromosomes as sister chromatids move to opposite poles in anaphase.
(2.82 MB MOV)

Video S2 Second example of a *Cdc20* ^−/H^ oocyte undergoing chromosome missegregation during meiosis I. Chromosomes (red) were marked by injection of H2B-mRFP prior to GVBD. Note the presence of lagging chromosomes as sister chromatids move to opposite poles in anaphase.
(2.65 MB MOV)
