# Supplementary figures and images for: Cdc20 Is Critical for Meiosis I and Fertility of Female Mice
Source: PLoS Genet. 2010 Sep 30;6(9):e1001147. doi: 10.1371/journal.pgen.1001147 (PMC2947999; doi:10.1371/journal.pgen.1001147)

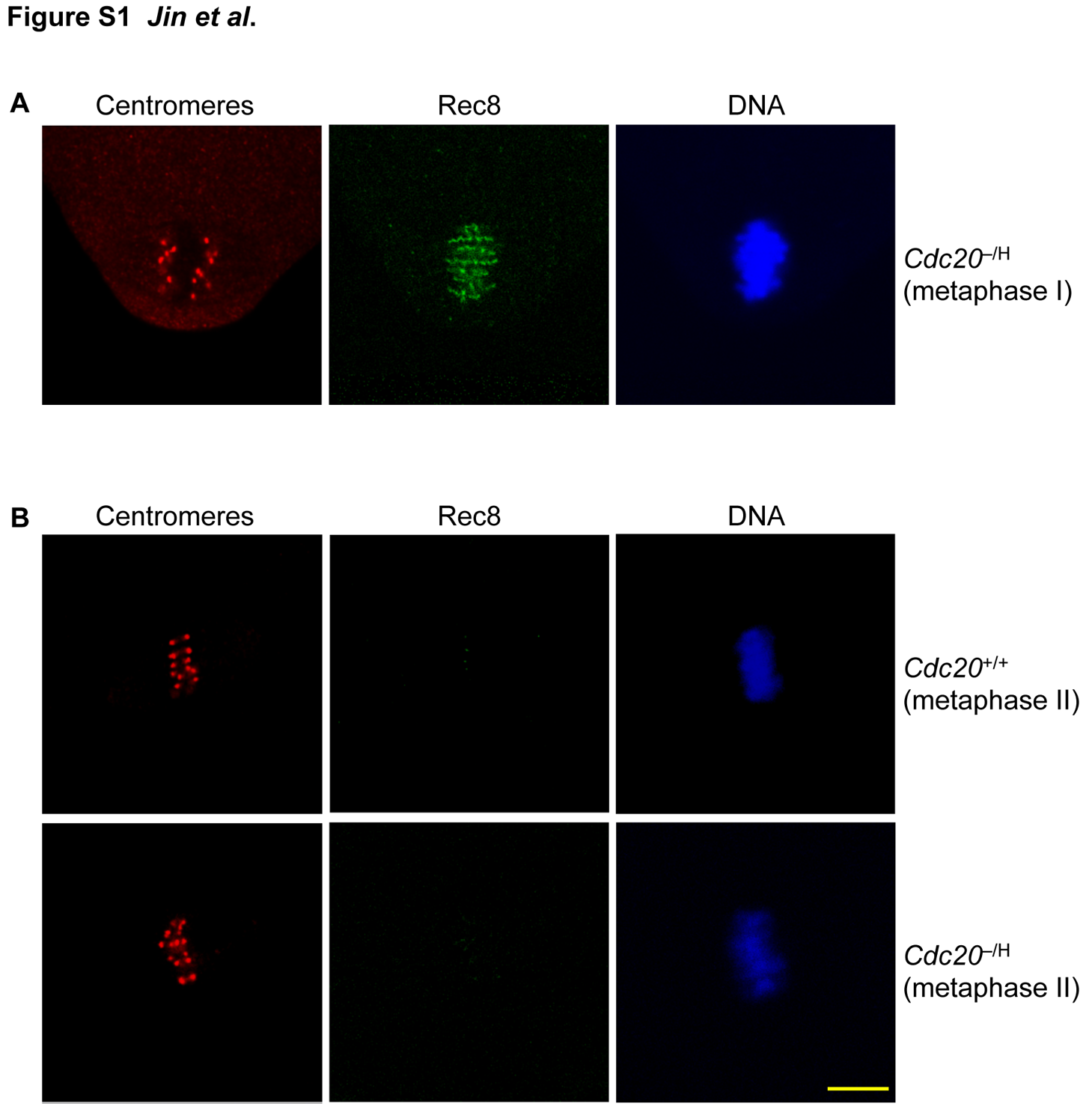

Supplement: Figure S1 — Chromosome missegregation in Cdc20 -/H primary oocytes does not seem to involve non-disjunction of bivalents. (A) A Cdc20 −/H primary oocyte in metaphase I stained for the meiotic cohesin component Rec8 [46], centromeres (ACA) and DNA (Hoechst). Note that Rec8 signals are localized along chromosome arms of bivalents. (B) Cdc20 +/+ and Cdc20 −/H primary oocytes were cultured until metaphase II arrest, collected and stained for Rec8, centromeres and DNA. Note that chromosome arms in metaphase II are negative for Rec8 irrespective of genotype, indicating the lack of bivalents. Scale bar represents 10 µm. (0.61 MB TIF) [file pgen.1001147.s001.tif]

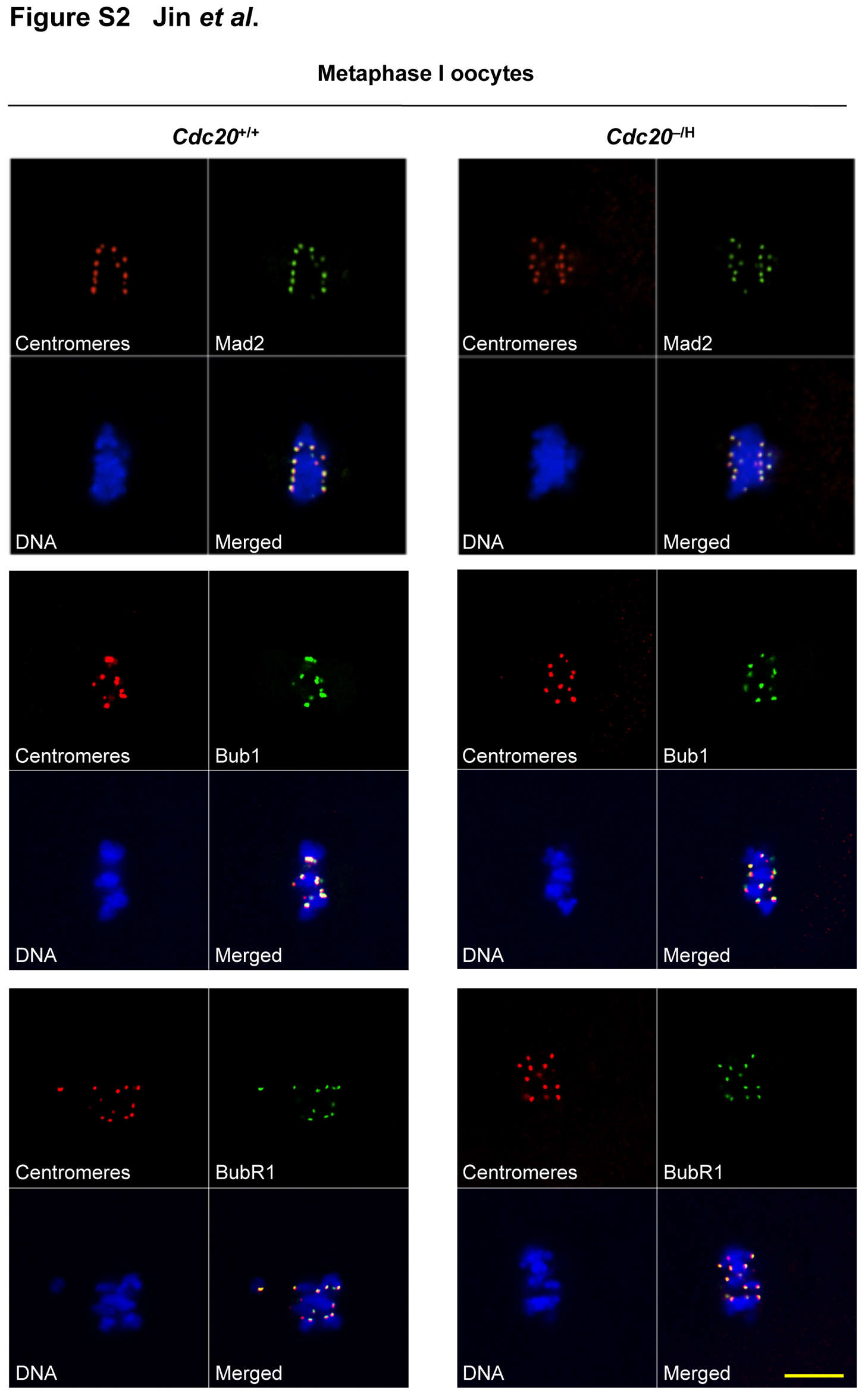

Supplement: Figure S2 — Mitotic checkpoint proteins properly localize to kinetochores of Cdc20 −/H primary oocytes. Cdc20 +/+ and Cdc20 −/H primary oocytes were harvested from ovaries and cultured until they had progressed to metaphase I (∼7 h after GVBD). Oocytes were fixed and immunostained for centromeres (ACA antibody) and either Bub1, BubR1 or Mad2. DNA was visualized by Hoechst staining. Scale bar represents 10 µm. (1.22 MB TIF) [file pgen.1001147.s002.tif]

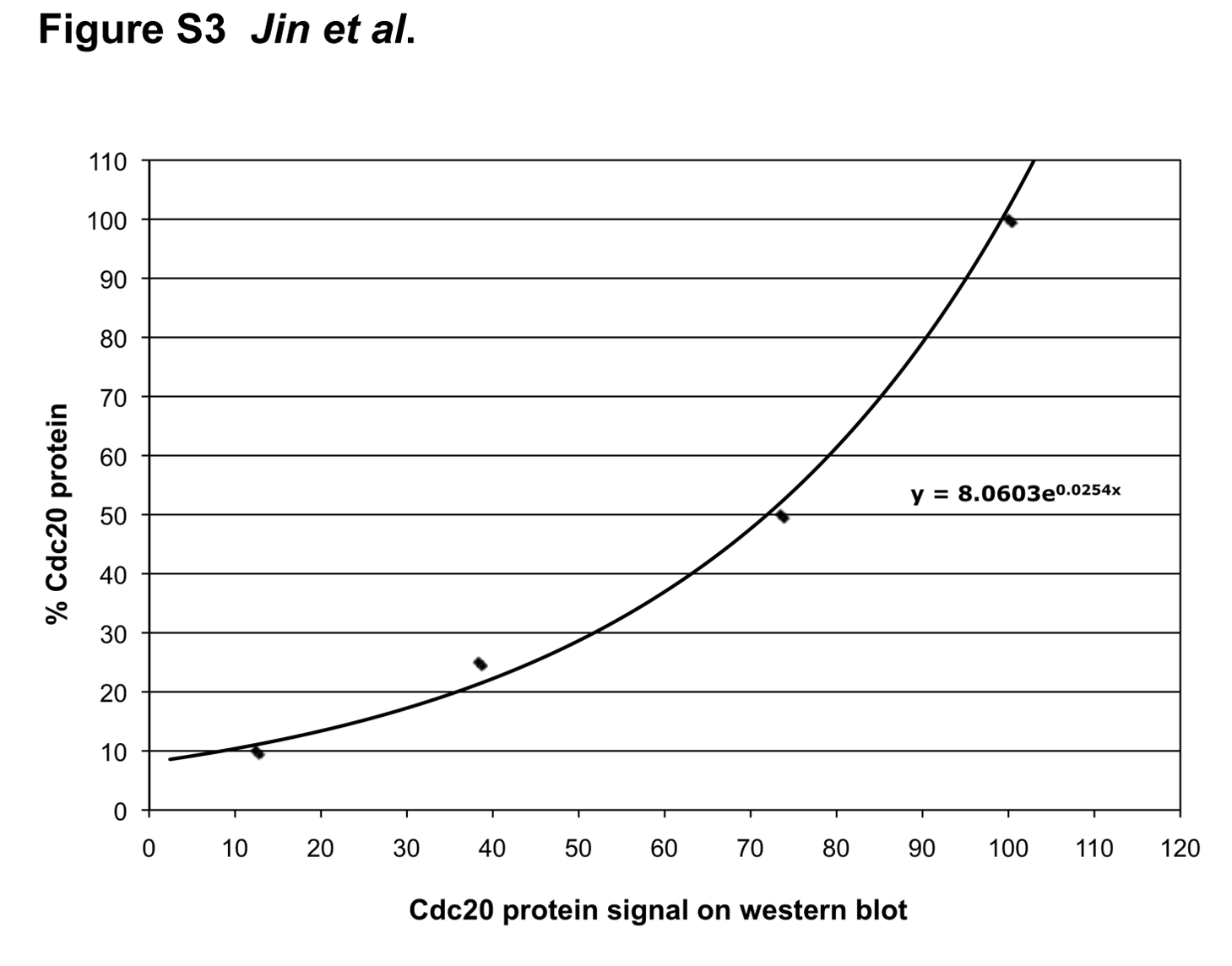

Supplement: Figure S3 — Percent Cdc20 protein plotted versus the average band intensity on western blots. (0.14 MB TIF) [file pgen.1001147.s003.tif]
